# Inter- and intra-species interactions between meat plant environmental bacteria and a non-biofilm-forming *Escherichia coli* O157:H7 strain in co-culture biofilms

**DOI:** 10.3389/fmicb.2024.1517732

**Published:** 2025-02-14

**Authors:** Jeyachchandran Visvalingam, Peipei Zhang, Xianqin Yang

**Affiliations:** Agriculture and Agri-Food Canada, Lacombe Research and Development Centre, Lacombe, AB, Canada

**Keywords:** *Escherichia coli*, *E. coli* O157, biofilm, meat-processing environmental bacteria, sanitation

## Abstract

This study evaluated the impact of meat-processing environmental bacteria (MPB) on biofilm formation by *Escherichia coli* O157:H7 in dual-species cultures. Biofilm development by 50 MPB and *E. coli* O157:H7 was assessed using crystal violet staining. Four MPB and *E. coli* O157:H7 combinations were evaluated further for viable cell numbers. A chlorinated alkaline agent and a quaternary ammonium-based agent were evaluated for their ability to remove biofilms. The *E. coli* O157:H7 strain was a non-biofilm former. In dual-species biofilms, if the companion MPB did not produce detectable biofilm, then the pairing did not produce measurable biofilms either. The interaction effect between MPB and *E. coli* O157:H7 was predominantly no-effect (neutral). Among the four MPB isolates tested by viable cell enumeration method, only generic *E. coli* genotype 136 reduced viable numbers of *E. coli* O157:H7 in dual-strain biofilm. Sequential treatment with cleaning and sanitizing treatment provided a better removal of biofilm than a single-agent treatment.

## 1 Introduction

*Escherichia coli* O157:H7 is one of the top-ranked foodborne pathogens globally because of the serious illness it often causes. Beef products have often been implicated in *E. coli* O157:H7 illnesses (CDC, 1993; Gaulin et al., 2015). Despite significant advancements in food safety standards (Zheng et al., 2023), outbreaks of *E. coli* O157:H7 linked with various food products including meat products continue to occur (Alberta Health Services, 2024; Smith et al., 2023; Coulombe et al., 2020). Studies have shown that *E. coli* O157:H7 can persist in food processing environments for extended periods of time (Varma et al., 2003; Williams et al., 2005). Even though the temperature during operations at beef processing plants may remain ≤10°C, it can exceed this limit during cleaning and sanitizing, as well as during non-operational hours (Mann and Brashears, 2006). During processing, *E. coli* O157:H7 can accumulate on beef processing surfaces along with meat debris and may remain in hard-to-reach areas due to ineffective cleaning and sanitizing (Youssef et al., 2013). As demonstrated in a recent study, *E. coli* O157 and other serogroups can grow in ground beef at temperatures between 5 and 15°C, mimicking a beef processing environment (Walker et al., 2023). This suggests that conditions at beef processing facilities may be conducive to the growth of *E. coli* O157:H7, leading to biofilm formation. Persistence of *E. coli* including *E. coli* O157:H7 in the food processing environments and subsequent contamination of food products has been attributed to biofilm formation rather than their resistance in planktonic form to biocides used in routine cleaning and sanitization (Wang et al., 2014; Yang et al., 2018).

Biofilms are structured aggregates of microbial cells that are encased by a moisture-rich matrix of self-generated extracellular polymeric substances (EPSs) (Hall-Stoodley and Stoodley, 2009; Yadav et al., 2020). Biofilm formation involves four discrete stages: initial reversible attachment of planktonic cells to a surface, irreversible attachment, microcolony growth with EPS production, and maturation and dissolution, which releases bacterial cells back into the planktonic state, enabling bacteria to colonize new niches (Van Houdt and Michiels, 2005). The entire biofilm formation process is a highly coordinated network of interactions of the bacterial cells and environmental cues, and consequently, the process and the characteristics of biofilms are affected by inherent characteristics of the bacterium as well as biotic and abiotic environmental factors (Chmielewski and Frank, 2003; Wang, 2019). The bacteria in biofilms are more resilient to environmental challenges due to their community structure providing protection by acting as a physical barrier, by chemical action involving charged interactions, and/or biological means involving the transfer of resistant elements or enzymatic action. As a result of these protective mechanisms, biofilm-embedded bacteria are much more tolerant to antimicrobial agents compared to their planktonic counterparts (Hall-Stoodley and Stoodley, 2009; Yadav et al., 2020; Van Houdt and Michiels, 2005). Sanitizers commonly used in processing facilities are largely ineffective against biofilms of *E. coli* (Wang et al., 2012).

Due to the role of biofilm formation in bacterial persistence, many studies have reported on the effects of various conditions on the biofilm of *E. coli* O157:H7 in both single and dual species (Wang et al., 2012; Nan et al., 2024; Wang et al., 2013). A recent study showed that a non-biofilm-forming strain of *E. coli* O157:H7 was at a similar level as a strong biofilm-forming *Salmonella* strain in mature biofilms when co-inoculated with a consortium of bacteria collected from post-sanitation equipment (Yang et al., 2023). In addition, many *E. coli* O157 strains lack biofilm-forming ability (Stanford et al., 2021). Meat-processing environmental bacteria (MPB) may influence the tolerance of *E. coli* O157:H7 to sanitizers in mixed biofilms (Dass et al., 2020). MPB consists of very diverse genera of bacteria and their influence on the biofilm formation of pathogens can vary significantly (Visvalingam et al., 2019; Fang et al., 2022). However, detailed studies examining the influence of MPB of diverse genera are lacking. Therefore, the objectives of this study were to evaluate how MPB belonging to different genera influence biofilm formation of a non-biofilm-forming *E. coli* O157:H7 strain and to evaluate the removal of mixed-species biofilms by commonly used sanitizers.

## 2 Materials and methods

### 2.1 Bacterial isolates

The MPB strains included in this study are listed in Supplementary Table S1. *E. coli* O157:H7 strain 1934 was originally recovered from beef and kindly provided by Dr. Alexander Gill (Health Canada, Ottawa, ON, Canada) and a non-biofilm former (Yang et al., 2023). Generic *E. coli* were recovered from beef cuts and trimmings as well as beef fabrication equipment at processing plants in previous studies and genotyped using multiple-locus variable-number tandem-repeat analysis (MLVA) (Yang et al., 2017a; Yang et al., 2015; Yang et al., 2017b). Genotypes that were recovered only from a single sampling visit were regarded as non-persistent, while those that were recovered from more than three sampling visits at the same facility were regarded as persistent. A total of 10 generic *E. coli* strains consisting of 5 genotypes selected at random from each of these 2 groups were included. In a previous study, 567 MPB were recovered from conveyor belts at a Canadian beef packing plant, which belonged to 40 genera (Wang et al., 2018). From these isolates, an isolate from each genus was randomly selected, consisting of 18 Gram-negative aerobic bacteria (GNA), 8 Gram-positive aerobic bacteria (GPA), 5 lactic acid bacteria (LAB), and 9 *Enterobacteriaceae* (ENT). In total, 50 bacterial isolates were included in the study, with 40 MPB and 10 generic *E. coli*.

### 2.2 Culture conditions and inoculum preparation

All bacterial isolates were stored at −80°C in half-strength Brain Heart Infusion broth (BHI; Oxoid, Mississauga, ON, Canada) containing 15% (v/v) glycerol (Fisher Scientific, Edmonton, AB, Canada), and working cultures were maintained on Trypticase Soy Agar (TSA; Oxoid) at 4°C with the monthly transfer. Lennox broth without salt (LB-NS, 10 g/L of tryptone and 5 g/L of yeast extract; Oxoid) was used for cultivating all bacterial isolates (Visvalingam et al., 2019), except for *Acinetobacter haemolyticus*, *Flavobacterium columnare*, *Pedobacter ginsengisoli*, and *Aerococcus urinaeequi*. These four isolates did not grow well in LB-NS (optical density at 600 nm < 0.1 after 120 h incubation at 25°C), so they were grown in BHI. A single colony from an agar plate was picked and inoculated into 10 mL of LB-NS or BHI and incubated in a shaking incubator operated at 80 rpm and 25°C until the stationary phase. Each bacterial culture grown in LB-NS was diluted 100-fold in the same media to obtain a bacterial suspension containing approximately 10^7^ CFU/mL of cells. For bacterial strains grown in BHI, a 1 mL portion of each bacterial suspension was centrifuged at 10,000 × *g* for 10 min at 4°C to pellet cells. The resulting pellet was re-suspended in 1 mL of LB-NS and diluted 100-fold in LB-NS. A total of 51 diluted suspensions were used as inoculum for the biofilm experiments below.

### 2.3 Monoculture, co-culture biofilm development, and quantification

Biofilms were grown using a device consisting of a 96-peg lid (Nunc Immuno TSP lid; Fisher) fitted to a 96-well round bottom microtiter plate (Nunc; Fisher). Inocula of 50 co-cultures were prepared by mixing an equal volume of *E. coli* O157:H7 and each of the 50 MPB inocula suspensions. A 160 μL of aliquot from each of the 51 strains or each of the 50 co-culture inocula was added to duplicate wells. Duplicate wells containing 160 μL of non-inoculated LB-NS were included as blank. Inoculated plates were fitted with pegged lids and incubated at 15°C for up to 6 days.

Biofilms formed on pegs were quantified using the previously described crystal violet (CV) staining method (Visvalingam et al., 2019). Briefly, after 2, 4, or 6 days of incubation, loosely attached planktonic cells were removed by successively placing pegged lid into two microtiter plates containing 160 μL of phosphate-buffered saline (PBS; Hardy Diagnostics, Santa Maria, CA, United States) per well. For each wash, pegs were incubated in PBS for 1 min at ambient temperature. Then, biofilms formed on pegs were stained for 20 min by placing the pegged lid into a microtiter plate with 160 μL of 0.1% (w/v) aqueous CV solution in each well, followed by rinsing off excess CV using two successive PBS washes as described above. The rinsed pegged lid was placed into a microtiter plate containing 180 μL of 80% (v/v) ethanol (Azer Scientific Inc., Morgantown, PA, USA) in each well for de-staining. After 20 min of de-staining, the pegged lid was replaced with a regular lid without pegs (Nunc microwell lid, Fisher) and absorbance of CV was determined at 570 nm (*A_570_*) using a POLARstar Omega microplate reader (BMG LABTECH GmbH, Ortenberg, Germany). Blank-corrected values generated by microplate data analysis software (BMG LABTECH) were used for analysis. Three independent experiments were performed for each monoculture and for each co-culture combination.

### 2.4 Enumeration of bacteria in biofilms

To understand how differences in biofilm-forming ability as determined by CV staining in comparison with that determined by enumerating viable bacterial cell numbers, a strong biofilm-forming *E. coli* genotype 136 (EC136), moderate biofilm-forming *Acinetobacter haemolyticus;* strong biofilm-forming *Sphingopyxis bauzanensis* and weak biofilm-forming *Carnobacterium maltaromaticum* were selected. Interaction between these strains and *E. coli* O157:H7 was also investigated. To avoid disrupting biofilm while removing pegs from lids, aseptically removed pegs from lids (from Nunc Immuno TSP lid) were used. For monoculture biofilm, 160 μL of EC136, *E. coli* O157:H7, *A. haemolyticus, S. bauzanensis,* or *C. maltaromaticum* inoculum was added into a 2-ml micro-centrifuge tube. Subsequently, an aseptically removed peg was placed into the inoculum to cover two-thirds of its length and incubated for 4 days as described above. To obtain co-culture biofilms, a total volume of 160 μL of EC136, *A. haemolyticus, S. bauzanensis,* or *C. maltaromaticum* and *E. coli* O157:H7 (80 μL each) were added to a 2-ml micro-centrifuge tube with a peg and incubated as described above. On day 4, the pegs were removed and each was washed twice using 160 μL of PBS. The washed pegs were each transferred to 15-ml centrifuge tubes containing 3 mL of 0.1% (w/v) peptone water and 0.3 g of glass beads (500 μm; BioSpec Products, Bartlesville, OK, USA) and vortexed at the maximum speed for 1 min. The resulting suspension was serially diluted in 0.1% peptone water and appropriate dilutions were spread-plated on selective agar plates. For the *E. coli* genotype 136 and *E. coli* O157:H7 combination, MacConkey agar (MAC; Oxoid,) and Sorbitol MacConkey agar supplemented with Cefixime-Tellurite (CT-SMAC; Oxoid) plates were used for enumeration of the total bacterial number and *E. coli* O157:H7, respectively. The *C. maltaromaticum* and *E. coli* O157:H7 combination was enumerated using All Purpose TWEEN^®^ agar (Oxoid) supplemented with 20 mg/L of nalidixic acid (APT-NA; Sigma-Aldrich, Oakville, ON, Canada) and CT-SMAC, respectively (Edima et al., 2007). For *A. haemolyticus, S. bauzanensis,* and *E. coli* O157:H7 combinations, TSA and CT-SMAC plates were used for enumeration of the total bacterial numbers and *E. coli* O157:H7, respectively. Inoculated MAC and CT-SMAC plates were incubated at 35°C for 24 h, while inoculated APT-NA and TSA plates were incubated at 25°C for up to 72 h. Then, plates containing 30 to 300 colonies were counted. Experiments were independently conducted twice, and three pegs were analyzed in each experiment.

### 2.5 Effects of a commonly used cleaner and sanitizer on biofilms

Meat-processing plants generally conduct cleaning and disinfection of the surfaces of the facility after daily operation. A commonly used chlorinated alkaline cleaner (Powerfoam Plus, Epsilon Chemicals Ltd., Edmonton, AB, Canada) and quaternary ammonium compound (QAC)-based sanitizer (E-San, Epsilon Chemicals Ltd), were selected to investigate the potential effects of the relevant chemicals used in this procedure on removing bacteria in biofilms. Dilutions of Powerfoam Plus (final concentration, 2.5%, v/v) or E-San (final concentration, 200 ppm) were prepared by following the manufacture’s instructions right before use. The combination of *E. coli* O157:H7 and *Acinetobacter haemolyticus*, which showed consistent synergistic interactions in biofilms, was included in the test.

**Figure 1:**
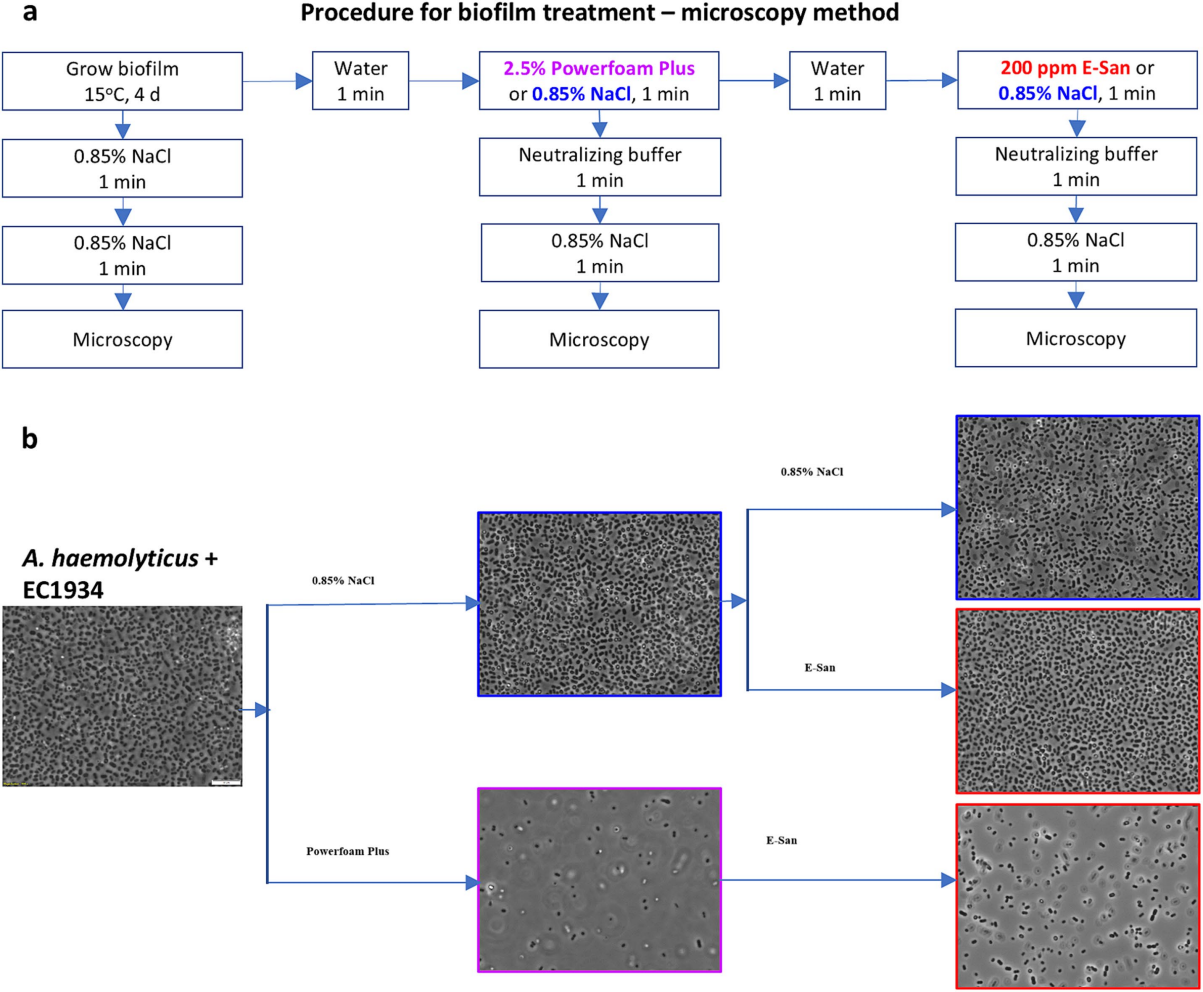
Effects of Powerfoam Plus and E-San in removing bacteria in biofilms (microscopy method). **(a)** The workflow of the treatments and **(b)** the representative images of treated biofilms under 1,000× magnification. Biofilms were formed by the co-culture of *Acinetobacter haemolyticus* and *E. coli* O157:H7 strain 1934 (EC1934) on cover glasses.

First, the microscopy method was used to examine the effects on biofilms. For this, biofilms were grown at 15°C for 4 days on cover glasses (18*18 mm; VWR, Edmonton, AB, Canada) placed in wells of a 12-well tissue culture plate (VWR) with each well containing 2 mL of mono- or co-culture of *E. coli* O157:H7 and/or *A. haemolyticus* on day 0. The biofilm-bearing cover glass was treated by being consecutively soaked in 2 mL of water, Powerfoam Plus/0.85% NaCl, water, and E-San/0.85% NaCl for 1 min (Figure 1a). Saline water (0.85% NaCl) was used as a control for Powerfoam Plus and E-San treatments. The treated cover glasses were washed twice using Difco™ Neutralizing Buffer (VWR, Edmonton, AB, Canada) and/or saline water to remove loosely attached bacterial cells (Figure 1a). The remained bacteria/biofilms on the cover glasses were examined using an Olympus light microscope BX53 equipped with a digital camera DP80 and cellSens imaging software at 1,000 × magnification.

**Figure 2:**
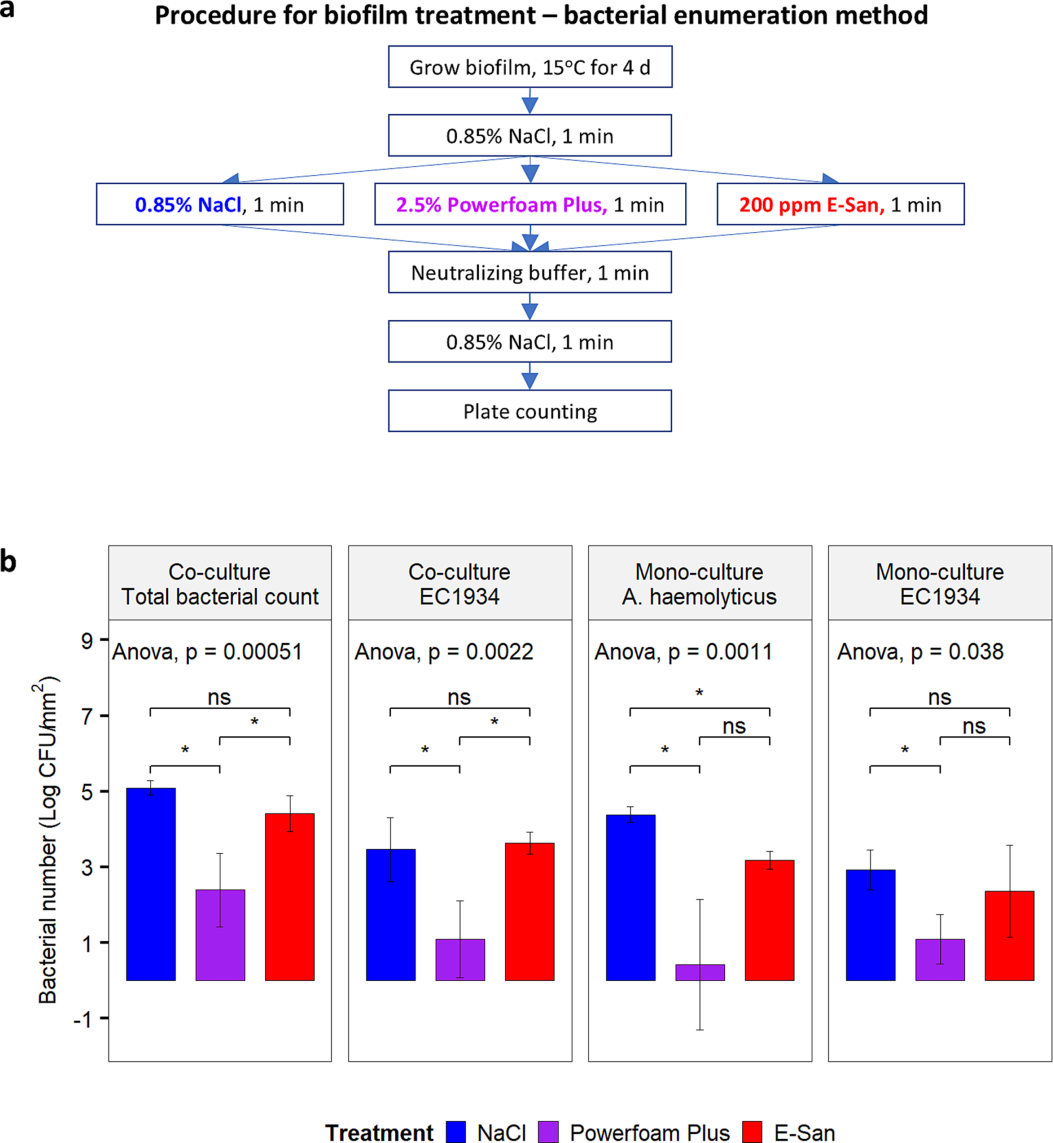
Effect of Powerfoam Plus and E-San in removing bacteria in biofilms (bacterial enumeration method). **(a)** The workflow of the test and **(b)** the comparison between Powerfoam Plus and E-San. Biofilms were formed by dual or mono culture of *Acinetobacter haemolyticus* and/or *E. coli* O157:H7 strain 1934 (EC1934) on pegs. Saline water (0.85% NaCl, w/v) was used as control. The error bars stand for standard deviations of three biological replicates. Treatment combination denoted by * indicate significant difference (*p* < 0.05).

In addition, viable bacterial cells were further enumerated for biofilms treated by E-San, Powerfoam Plus, and saline water (control), respectively. For this purpose, biofilms were developed at 15°C for 4 days on detached pegs in a 96-well plate with each well containing 160 μL of mono- or co-culture. The biofilms were treated by following the procedure shown in Figure 2a. The number of *E. coli* O157:H7, *A. haemolyticus,* or total bacteria in treated biofilm was enumerated as described above.

### 2.6 Data analysis

Mean *A_570_* from CV staining was calculated. The cutoff value for biofilm formation (*A_570C_*) was calculated using the equation: *A_570C_ =* Mean *A_570_* of blank +3 × standard deviation of *A_570_* for blank (Stepanović et al., 2007). Based on *A_570_* values, strains were classified into four categories: non-biofilm former, weak, moderate, or strong biofilm former (Table 1). The interactions between two bacterial strains in a biofilm were regarded as synergistic, with no effect or antagonistic if the *A_570_* value of dual culture is larger than, equals to, or smaller than the higher of the *A_570_* values of the two relevant monocultures (Ren et al., 2015; Ren et al., 2014). Bacterial counts and *A_570_* values were analyzed using a one-way ANOVA with the help of GraphPad Prism 10 (GraphPad Software, Boston, MA, United States). The Tukey’s test was used to assess pairwise differences between means, with a significance level of *p* ≤ 0.05 and mean values were presented with standard error of means. Two-way ANOVA was performed to analyze the effect of treatment and culture combinations on the bacterial numbers in the biofilm treated with different cleaning and sanitizing agents.

**Table 1:** Classification of biofilm formation based on scoring system modified from Stepanović et al. (2007).

Category	Score	*A_570_* limits	Maximum *A_570_* limit
Non-biofilm former	0	*A_570_* < 0.21	*A_570C_ = A_570_* of blank +3 × standard deviation of *A_570_* for blank = 0.21
Weak	1	0.21 < *A_570_ ≤* 0.42	2* A_570C_
	2	0.42 < *A570* ≤ 0.82	4* *A_570C_*
Moderate	3	0.82 < *A570* ≤ 1.68	8* *A_570C_*
	4	1.68 < *A570* ≤ 3.36	16* *A_570C_*
Strong	5	3.36 < *A570* ≤ 6.72	32* *A_570C_*
	6	6.72 < *A570* ≤ 13.44	64* *A_570C_*

## 3 Results

### 3.1 Mono- and dual-culture biofilms

**Figure 3:**
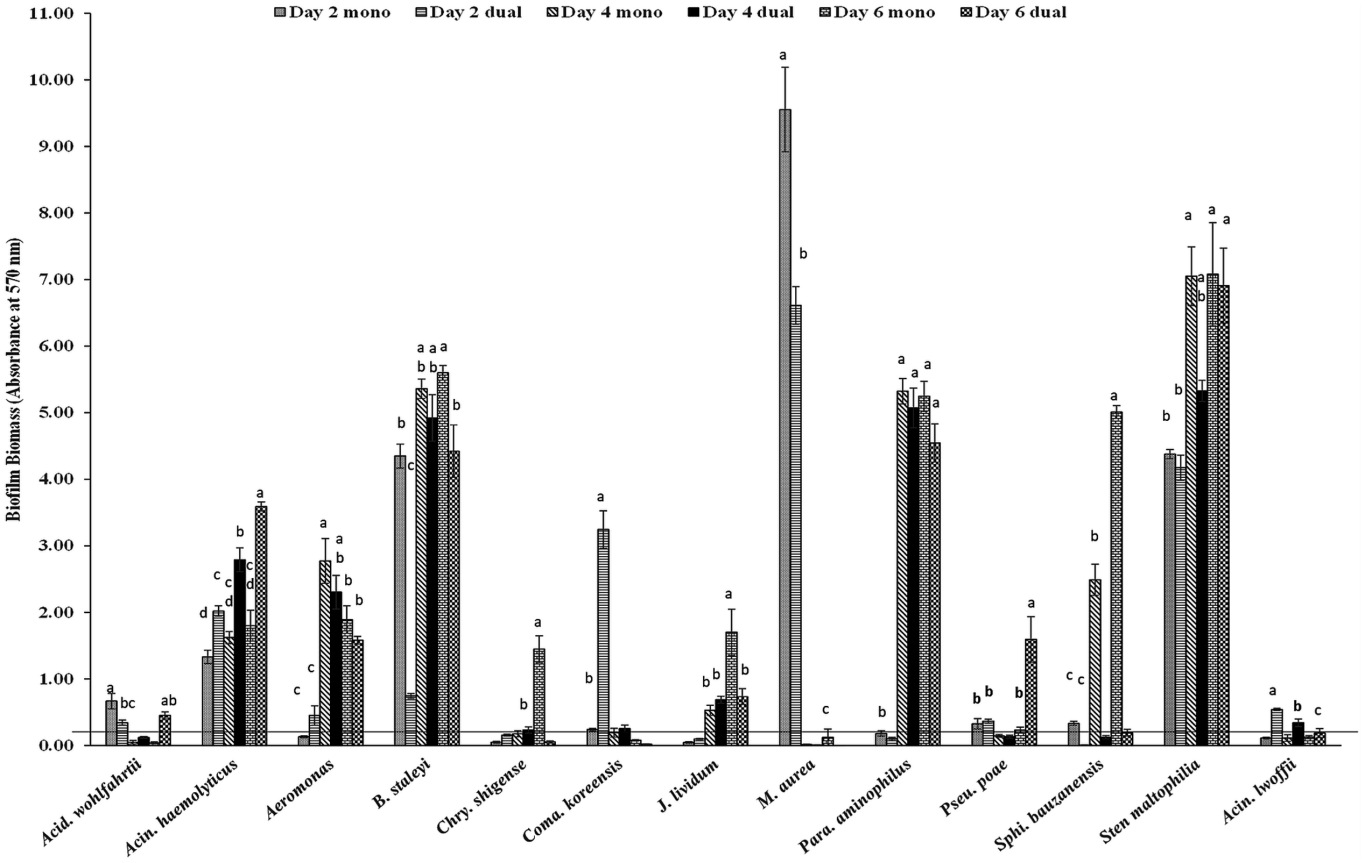
Biofilm formation of *E. coli* O157:H7 and meat plant Gram-negative aerobes (GNA) recovered from a meat packing plant in mono or dual species. Mean values identified with different letters are significantly different (*p < 0.05*). Horizontal line indicates the cutoff for biofilm former.

**Figure 4:**
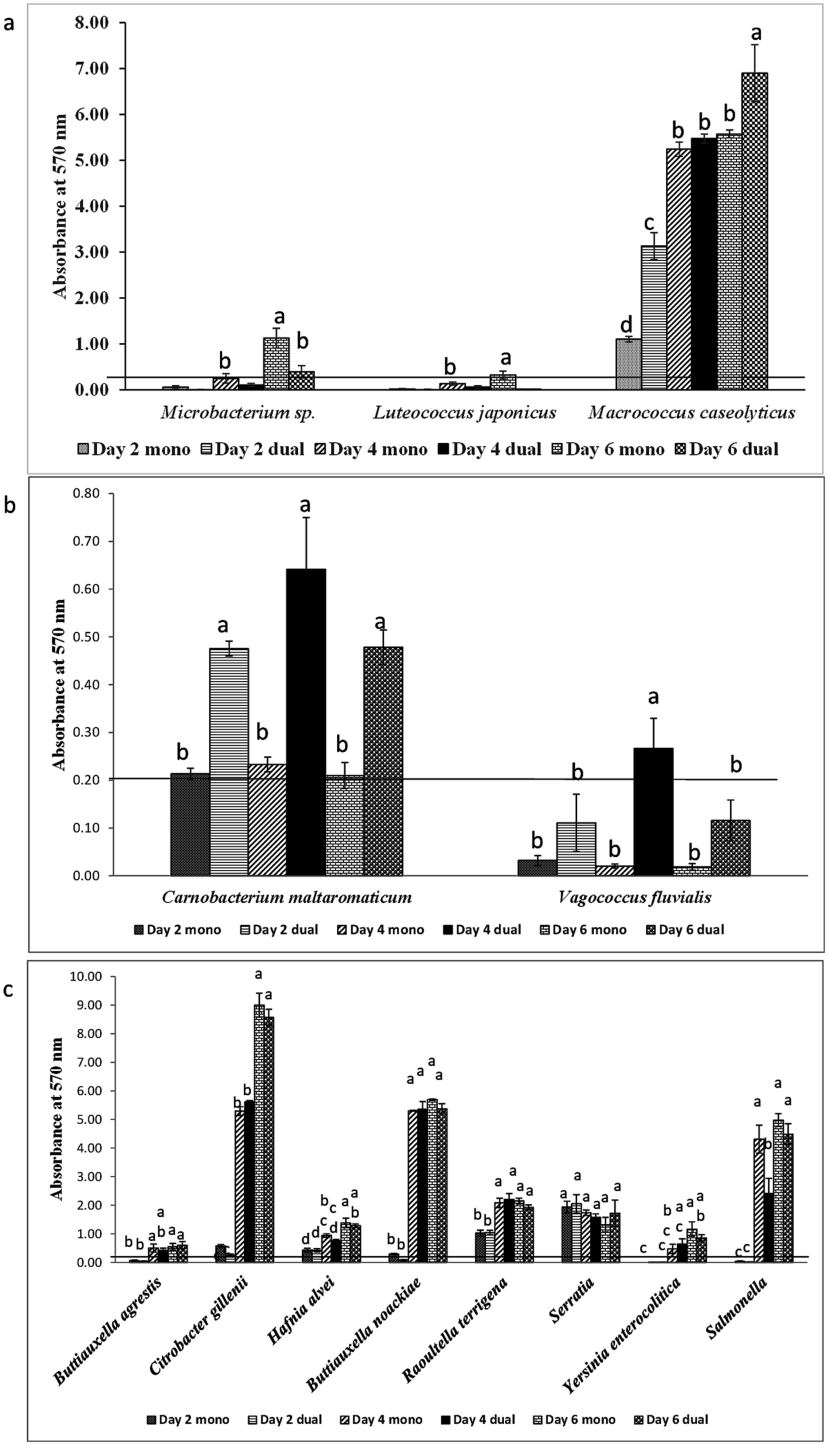
Biofilm formation of **(a)** *E. coli* O157:H7 and meat plant Gram-positive aerobes (GPA), **(b)** *E. coli* O157:H7 and lactic acid bacteria (LAB), and **(c)** *E. coli* O157:H7 and *Enterobacteriaceae* (ENT) recovered from a meat packing plant in mono or dual species. Mean values identified with different letters are significantly different (*p < 0.05*). Horizontal line indicates the cutoff for biofilm former.

Among 18 GNA and 8 GPA, 61.1% and 25–37.5% of isolates formed monoculture biofilm between days 2 and 6, respectively (Table 2). The majority of those GNA and GPA strains formed weak or moderate biofilm with the exception of the GNA stains *Brevundimonas staleyi*, *Massilia aurea*, and *Stenotrophomonas maltophilia*, and the GPA strain *Macrococcus caseolyticus*, all of which formed strong biofilm at one or more sampling points (Figures 3, 4a). The *E. coli* O157:H7 strain was a non-biofilm former with *A_570_* values below the cutoff value of 0.21. When it was co-cultured with GNA, more GNA formed biofilm on days 2 and 4 than on day 6 (Table 2). Unlike GNA, fewer GPA strains formed co-culture biofilm on days 2 and 6 than on day 4.

**Table 2:** Frequency of biofilm formation by beef packing plant environmental isolates and *Escherichia coli* O157:H7 in mono- or co-cultures.

Bacteria group	Number of isolates	Percent of isolates formed biofilm					
		Day 2		Day 4		Day 6	
		Monoculture	Co-culture	Monoculture	Co-culture	Monoculture	Co-culture
GNA	18	61.1	66.7	61.1	66.7	61.1	55.6
GPA	8	25.0	12.5	37.5	37.5	37.5	25.0
LAB	5	20.0	20.0	20.0	40.0	20.0	20.0
ENT	9	66.7	55.6	88.9	88.9	88.9	88.9
GEC	10	80.0	80.0	80.0	80.0	80.0	80.0

**Figure 5:**
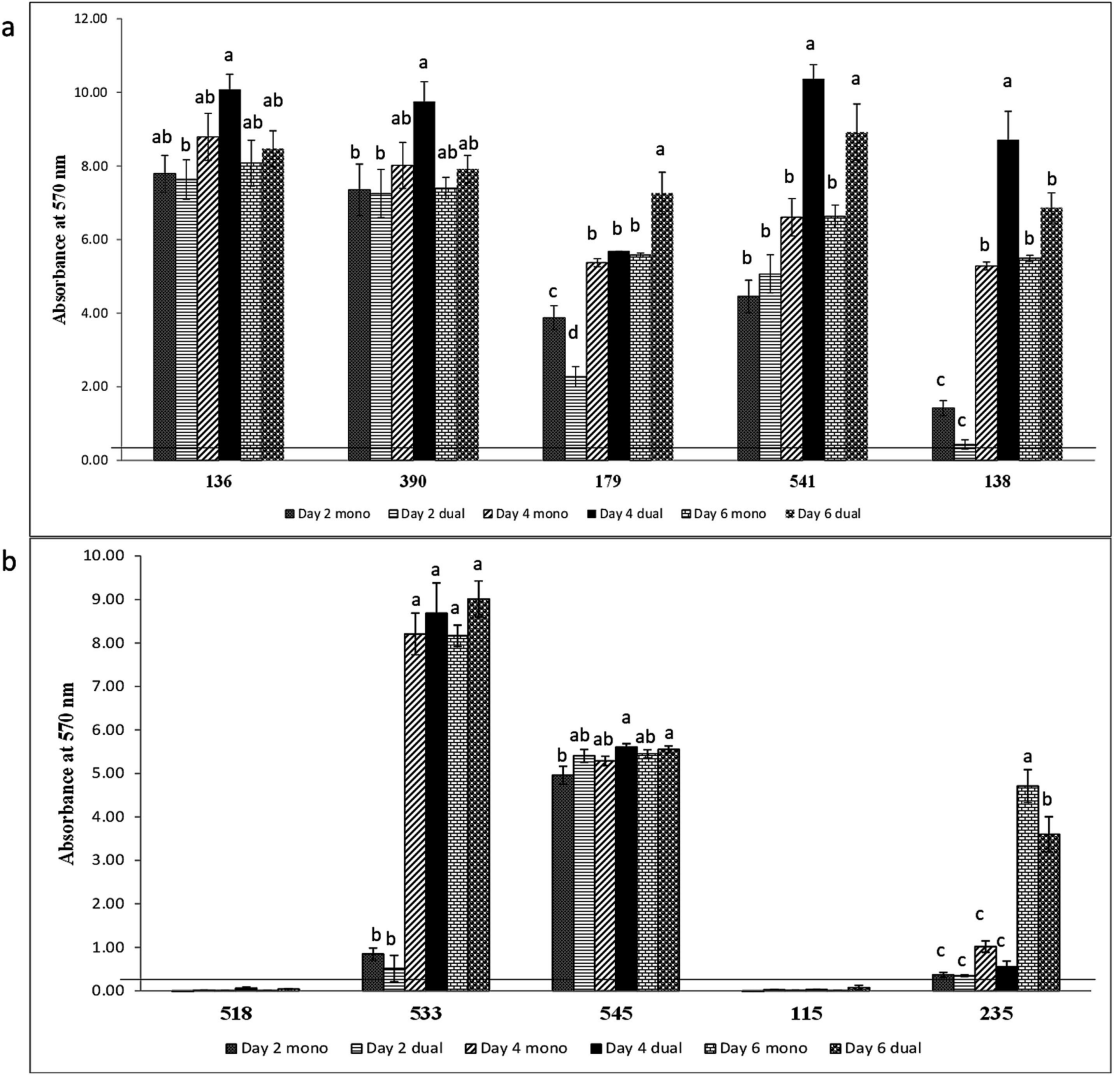
Biofilm formation of **(a)** *E. coli* O157:H7 and persistent generic *E. coli* and **(b)** *E. coli* O157:H7 and non-persistent generic *E. coli* recovered from a meat packing plant in mono or dual strain. Mean values identified with different letters are significantly different (*p < 0.05*). Horizontal line indicates the cutoff for biofilm former.

Of the five LAB isolates tested, only *C. maltaromaticum* formed measurable, but weak biofilms (Figure 4b). When co-cultured with *E. coli* O157:H7, only *C. maltaromaticum* and *Vagococcus fluvialis* formed biofilms (Figure 4b). At day 2, 66.7% of ENT strains formed biofilms which reached 88.9% by day 4 with no further increase by day 6 (Table 2), with *Citrobacter gillenii*, *Buttiauxella noackiae*, and *Salmonella* sp. being strong biofilm formers (Figure 4c). When co-cultured with *E. coli* O157:H7, all strains that formed monoculture biofilms also formed dual-species biofilms by day 4 (Figure 4b). As previously described (Visvalingam et al., 2017), biofilm formation of GEC strains remained at 80% between days 2 and 6 (Table 2). All persistent GEC genotypes and three of the non-persistent GEC genotypes formed biofilms. In general, persistent genotypes formed stronger biofilm than non-persistent ones, more so on day 2 than on days 4 and 6 (Figures 5a, b). All persistent and non-persistent strains that formed mono-culture biofilms also formed dual-culture biofilms with *E. coli* O157:H7.

**Figure 6:**
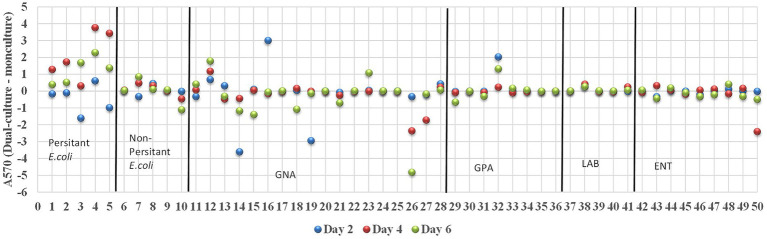
Interactions between *E. coli* O157:H7 and MPB in biofilms. The interactions were determined as synergistic, antagonistic, and neutral if the A570 (dual-culture –monoculture) is greater than, smaller than and equals to zero, respectively. Strains of 1–5, 6–10, 11–28, 29–36, 37–41, and 42–50 are groups of persistent *E. coli*, non-persistent *E. coli*, GNA, GPA, LAB, and ENT, respectively.

Biofilm-forming interaction was classified as no-effect, synergy, and antagonism based on *A_570_* values observed between monoculture and dual-culture biofilms. Overall, the interactions between MPB and *E. coli* O157:H7 in their respective dual-culture biofilms were mostly no-effect (Figure 6). Consistent interactions with *E. coli* O157:H7 in co-culture biofilms were observed for the GNA isolates *B. staleyi* and *S. bauzanensis* (antagonistic; #14 and #26), and *A. haemolyticus* (synergistic; #12) during the study period (Figures 3, 6). GPA isolates *Microbacterium* sp., (#29) and *Macrococcus caseolyticus* (#32) formed antagonistic and synergistic relationship, respectively (Figures 4a, 6). Interestingly, with the commonly regarded biocontrol agents, lactic acid bacteria, only synergistic effects were observed (*C. maltaromaticum*, #38; *V. fluvialis*, #41). None of the ENT isolates had interactions in co-culture biofilms except for *Salmonella* sp. (#50) which was antagonistic. Persistent *E. coli* isolates mainly showed synergistic interactions when forming biofilms with *E. coli* O157:H7, while non-persistent isolates showed no effect.

### 3.2 Changes in viable cell numbers in mono-species and dual-species biofilms

EC136, *A. haemolyticus*, *S. bauzanensis*, and *C. maltaromaticum* selected based on their biofilm-forming ability and interactions with *E. coli* O157:H7 were further assessed by enumeration in single and dual-culture biofilms with *E. coli* O157:H7 (Table 3). In monoculture, the number of viable *E. coli* O157:H7 cells was 7 log CFU/peg. The viable cell numbers of *E. coli* O157:H7 did not differ significantly (*p > 0.05*) when it formed dual-species biofilms with *A. haemolyticus, S. bauzanensis*, or *C. maltaromaticum*. In contrast, viable numbers of *E. coli* O157:H7 were significantly lower (*p < 0.05*) when it formed biofilm with EC136. Viable numbers of EC136 and *C. maltaromaticum* did not differ between monoculture and dual-culture biofilms. Viable numbers of *A. haemolyticus* were significantly higher in dual-species biofilm containing *E. coli* O157:H7 than its monoculture biofilm while that of *S. bauzanensis* was lower (*p < 0.05*) in dual species than in its monoculture biofilms.

**Table 3:** Changes in number of viable *Escherichia coli* O157:H7 and meat plant environmental isolates in mono- and co-culture biofilms formed on polystyrene pegs incubated at 15°C for 4 days.

Bacterial strains	Biofilm type	Viable numbers (log CFU per peg)*
*E. coli* O157:H7	Monoculture	7.05 ± 0.22^X^
*E. coli* O157:H7	With *E. coli* genotype 136	5.53 ± 0.23^Y^
	With *A. haemolyticus*	7.00 ± 0.17^X^
	With *S. bauzanensis*	6.97 ± 0.30^X^
	With *C. maltaromaticum*	6.92 ± 0.26^X^
*E. coli* genotype 136	Monoculture	7.70 ± 0.20^A^
	Dual culture	7.56 ± 0.20^A^
*A. haemolyticus.*	Monoculture	6.29 ± 0.15^B^
	Dual culture	7.14 ± 0.23^A^
*S. bauzanensis*	Monoculture	7.24 ± 0.16^A^
	Dual culture	≤5.50^B§^
*C. maltaromaticum*	Monoculture	6.81 ± 0.12^A^
	Dual culture	7.03 ± 0.12^A^

*Statistical tests were performed for each genus separately. Mean values denoted by the same superscript letters for each genus were not significantly different (*P* > 0.05). ^§^Yellow colonies that are typical for *Sphingopyxis* sp., were not detected at plated dilutions.

### 3.3 Effects of a commonly used cleaner and sanitizer on biofilms

Due to the synergistic effect between *A. haemolyticus* and *E. coli* O157:H7, this combination was included to test the effects of a commonly used cleaner and sanitizer in removing biofilms or bacteria in biofilms. Microscopic observation of dual- (Figure 1b) and mono-culture biofilms (Supplementary Figure S1) showed an unnoticeable effect by E-San treatment alone. In contrast, Powerfoam Plus treatment alone removed a large portion of bacteria in both types of biofilms. The sequential treatment of Powerfoam Plus and E-San was more effective than Powerfoam Plus alone for mono-culture biofilms (Supplementary Figure S1), however, no difference was noticed for dual-culture biofilms (Figure 1b). Further comparison of Powerfoam Plus with E-San by enumerating bacteria in treated biofilms showed that non-significant but numerically stronger (total bacteria in co-culture biofilms; *E. coli* O157:H7 in mono-culture biofilms) or statistically significant stronger effects (*E. coli* O157:H7 in dual-culture biofilms; *A. haemolyticus* in mono-culture biofilms) by Powerfoam Plus in removing bacteria (1–3 log CFU) in biofilms than E-San (Figure 2b). No significant difference was observed between E-San and control (saline water).

## 4 Discussion

Most bacteria predominantly reside in the form of biofilms in all environments, most likely with companion bacteria. The characteristics of mixed-species biofilm community can significantly be influenced by unavoidable direct and indirect interactions between different species (Penesyan et al., 2021; Burmølle et al., 2014). This helps bacteria survive diverse environmental challenges, including conditions experienced in food processing environments (Wang et al., 2013; Visvalingam et al., 2019; Habimana et al., 2010) and potentially serve as reservoir for product contamination (Wang, 2019). Therefore, understanding their interactions and growth is essential for controlling biofilms in food processing environment and prevent potential cross contamination. This study evaluated the impact of meat plant isolates, including both persistent and non-persistent *E. coli* and MPB, on the biofilm formation of *E. coli* O157:H7 at 15°C. Although beef processing facilities typically operate at temperatures <10°C, temperatures can rise to 15°C during non-operational hours or during cleaning and sanitizing processes (Youssef et al., 2013; Yang et al., 2018). If sanitation protocols fail to completely remove microbes and food residues, these higher temperatures could facilitate microbial growth. To accurately assess this potential risk, the biofilm experiments in this study were conducted at 15°C. At this temperature, *E. coli* could establish mature biofilms by 4 days (Visvalingam et al., 2017) and sanitizer resistance may increase with the age of biofilms (Chavant et al., 2004). In addition, many of the MPB isolates used in this study grew at a much slower rate than *E. coli* O157:H7, with some strains taking up to 120 h to reach required cell density during inoculum preparation. Thus, a 6-day incubation time was chosen to properly assess the influence of MPB on biofilm formation. The impact of companion bacteria on the non-biofilm-forming strains of pathogens is a rather underexplored area even though these pathogenic strains could also achieve persistence status by hitchhiking with others (Yang et al., 2023; Visvalingam et al., 2019).

The inconsistency between biomass measurement and viable cell number enumeration for assessing biofilm formation of the *E. coli* O157:H7 strain has also been noted in a previous study where motile *E. coli* O157:H7 (02:0627) and non-motile *E. coli* O157:NM (02:1840) did not form detectable biofilm by CV assay while approximately 7 Log CFU viable cells were adhered to surface (Visvalingam et al., 2017). Those two latter *E. coli* O157 strains did not have the ability to produce curli and cellulose, two major components in EPS. Detectable biofilm formation by *E. coli* O157 and non-157 Shiga toxin-producing *E. coli* strains was highly correlated to the production of curli and cellulose (Wang et al., 2012; Fang et al., 2022). As EPSs take up 90% biomass of biofilms (Toyofuku et al., 2016), it is inferable that lack of curli or cellulose may have led to this inconsistency between viable cell enumeration and the commonly used CV staining method.

In dual-species biofilms, if a companion MPB did not produce detectable biofilm then the *E. coli* O157:H7-MPB pairing did not produce measurable biofilms either. A notable exception from this was the paring of *E. coli* O157:H7 and LAB isolate *V. fluvialis*. The *V. fluvialis* isolate did not form biofilms on its own at any given point, but formed measurable biofilms with the *E. coli* O157:H7 strain at day 4. Interaction of microorganisms in mixed-species biofilms involves interference with quorum sensing and production of secondary metabolites or toxins that promote or inhibit the growth of companion microorganisms (Habimana et al., 2010; Jahid et al., 2018; Chorianopoulos et al., 2010). Based on biomass, the LAB isolates *C. maltaromaticum* and *V. fluvialis* were synergistic with *E. coli* O157:H7. A similar interaction was observed with *C. maltaromaticum* and *S.* Typhimurium in another study (Visvalingam et al., 2019). Further analysis of viable numbers in the dual-species biofilm of *E. coli* O157:H7 and *C. maltaromaticum* showed the viable numbers for either of these organisms did not differ from their monoculture biofilm counterparts. This divergence could stem from an elevated production of EPSs rather than an increase in viable numbers within the dual-species biofilms. Enhanced EPS production has been documented in mixed-species biofilms, such as those formed by *Salmonella* and *Pseudomonas aeruginosa* or indigenous lettuce microbiota (Jahid et al., 2014; Pang et al., 2017). Additionally, some of the viable cell populations may not be accurately represented by standard enumeration methods, as many species can enter a viable but non-culturable (VBNC) state (Leriche and Carpentier, 1995). In contrast to what was observed in *E. coli* O157:H7-*C. maltaromaticum,* synergistic interaction observed between *A. haemolyticus* and *E. coli* O157:H7 by biomass measurement using CV assay coincided with higher viable numbers of *A. haemolyticus* in dual-species biofilm but not *E. coli* O157:H7. A synergistic effect between a meat plant isolate of *A. calcoaceticus* and an *E. coli* O157:H7 strain has been noted in a study by Habimana et al. (2010). Previously, Makovcova et al. (2017) observed that when *Staphylococcus aureus* and *S. enterica* or *E. coli* have formed dual-species biofilm, only *S. aureus* numbers increased in dual-species biofilm compared to its monoculture counterpart.

Interestingly, biomass measurement of EC 136-*E. coli* O157:H7 co-culture biofilm showed no effect to slight synergistic effect without a significant increase in EC 136 or *E. coli* O157:H7 viable numbers. In fact, the numbers of *E. coli* O157:H7 significantly decreased in co-culture biofilm compared to its monoculture biofilm. The ability of EC 136 and *E. coli* genotype 533 to reduce viable numbers of motile *E. coli* O157:H7 (02:0627) or non-motile *E. coli* O157:NM (02:1840) in their respective co-culture biofilm was previously reported (Visvalingam et al., 2017). Different from the intra-species antagonistic effect, antagonistic interaction observed between *E. coli* O157:H7 and *S. bauzanensis* resulted in both a reduction in biofilm biomass and a reduction in viable numbers of *S. bauzanensis* compared to its monoculture biofilm while *E. coli* O157:H7 numbers remained similar between dual-species and monoculture biofilm. This difference in the biomass assessment and cell-number assessment may have resulted from the lack of curli or cellulose production of the *E. coli* O157:H7 strain or due to excessive EPS production by the dominating *E. coli* strain. It is conceivable that observed antagonistic interaction between generic *E. coli* and *E. coli* O157:H7 could result from their competing nutritional needs or production of inhibitory substances such as colicin (Schamberger and Diez-Gonzalez, 2004; Setia et al., 2009). Nevertheless, the synergistic effect of GEC, especially the persisting ones, with *E. coli* O157 is concerning, suggesting companion bacteria have to be taken into account when examining the persistence of pathogens.

Taken together, the synergistic or antagonistic interactions observed in mixed-species/strain biofilms as measured by biomass, do not always indicate an increase or decrease in viable numbers, or *vice versa*. When it comes to contamination control in meat-processing plants, both the biomass and the viable cell numbers within biofilms are significant factors that may impact the disinfectant tolerance of pathogens. Therefore, the assessment of mixed-species biofilms associated with the food processing environment requires a combination of assessment methods.

Previous studies conducted with QAC-based sanitizer E-San and Perox-E PLUS against *S.* Typhimurium and MPB mixed-species biofilm showed limited effectiveness in reducing viable cells in biofilms and in an MPB-dependent manner when there was any effect (Visvalingam et al., 2019). At the in-use concentration of 200 ppm, E-San reduced the numbers of *S.* Typhimurium in its mono-culture biofilms and *J. lividum* or *Serratia* co-culture biofilms by 1.08, 1.57, and 1.97 log units, respectively. However, it was entirely ineffective against *S.* Typhimurium in co-culture biofilms with *A. haemolyticus*, *M. phyllosphaerae*, or *P. helvolus*. Survival of *E. coli* O157:H7 in mixed-species biofilm when treated with QAC at 300 ppm has also been reported to be dependent on its companion strains (Wang et al., 2013; Dass et al., 2020). In the present study, E-San was largely ineffective against *E. coli* O157:H7 in either mono or co-cultures with *A. haemolyticus*. In contrast, Powerfoam Plus showed a much stronger effect in removing bacteria in the dual- or mono-culture biofilms. Test conducted with a chlorinated alkaline cleaner previously showed it can remove 99% EPSs of *P. putida* biofilm but this study did not report any changes in viable numbers (Antoniou and Frank, 2005). Removal of *Listeria monocytogenes* biofilm was evaluated after treatment with various alkaline or acidic cleaning agents including chlorinated alkaline cleaner and it was found single treatment using all tested cleaners yielded 1–2 log CFU reduction in viable numbers (Fagerlund et al., 2020).

In conclusion, the non-biofilm-forming *E. coli* O157:H7 formed biofilms with diverse MPB in an MPB strain-dependent manner. When an MPB strain did not form measurable biofilm on its own as determined by biomass, *E. coli* O157:H7 did not form dual-species biofilm with that isolate except for *V. fluvialis.* Interaction between *E. coli* O157:H7 and MPB was pre-dominantly neutral when assessed by biofilm mass. The present study offers a glimpse into the interactions of a non-biofilm-forming *E. coli* O157:H7 strain with meat-processing environmental bacteria. Given that most Shiga toxin-producing *E. coli* lack the ability to form biofilms, when assessed by the CV staining method, and they may also achieve persistence through hitchhiking with others, further study to explore how those strains behave in multi-species setting would benefit the eventual control of these pathogens in food processing environment. Sequential treatment of Powerfoam Plus and E-San provided a better reduction in biofilm than treatment with one of those agents for monoculture biofilms. Powerfoam Plus alone achieved more than 2.7 log reduction of viable cells in dual-species biofilms.

## Data Availability

The raw data supporting the conclusions of this article will be made available by the authors, without undue reservation.
