# Supplementary figures and images for: Inter- and intra-species interactions between meat plant environmental bacteria and a non-biofilm-forming Escherichia coli O157:H7 strain in co-culture biofilms
Source: Front Microbiol. 2025 Feb 14;15:1517732. doi: 10.3389/fmicb.2024.1517732 (PMC11867959; doi:10.3389/fmicb.2024.1517732)

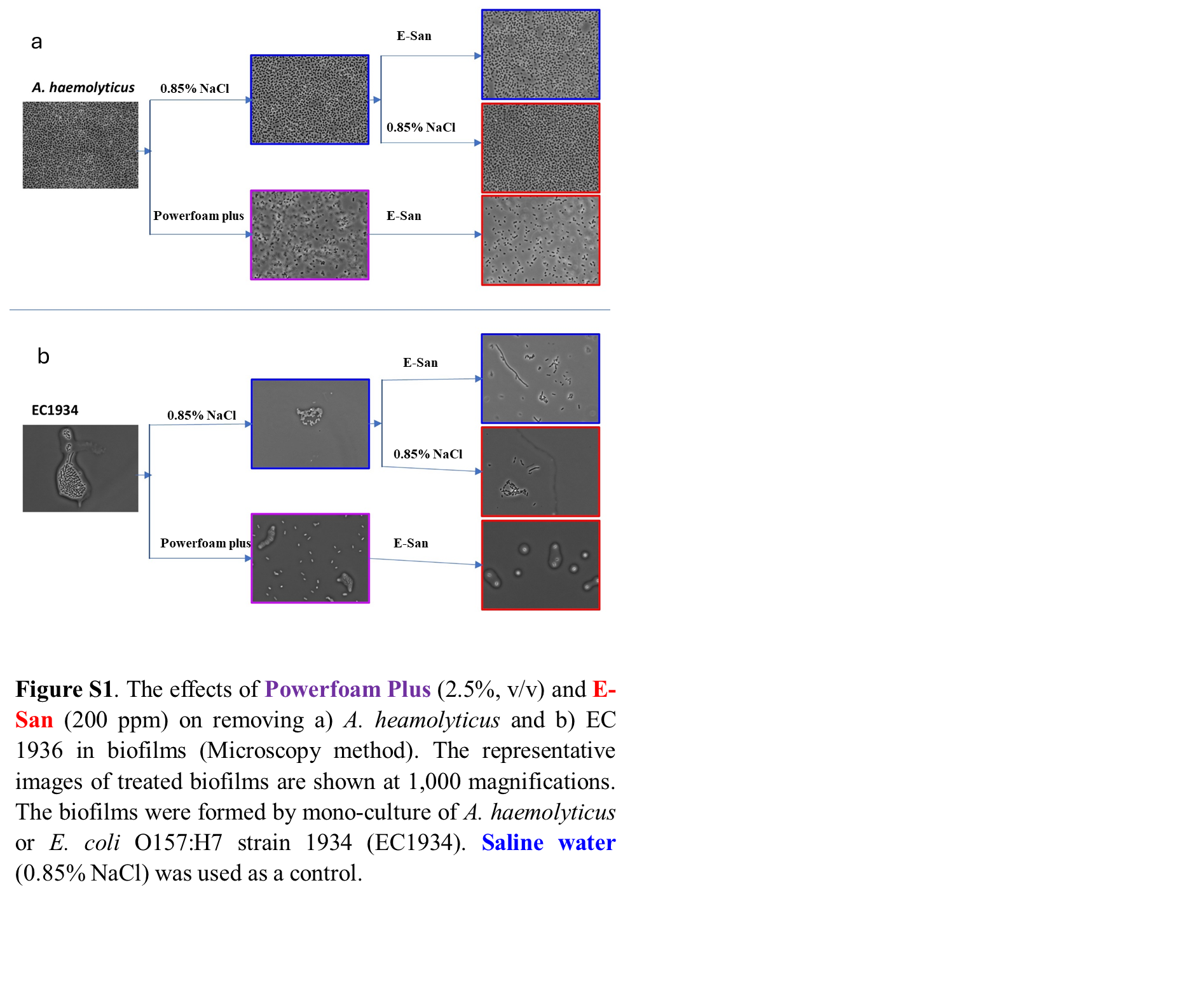

Supplement: Supplementary file 2 [file Image_1.tif]
